# The declaration of lima on pain in childhood

**DOI:** 10.1097/PR9.0000000000001055

**Published:** 2022-12-20

**Authors:** Jordi Miró, Marco A. Narváez, Enrique Orrillo, Pablo Ingelmo, João Batista S. Garcia

**Affiliations:** a Universitat Rovira i Virgili, Catalonia, Spain; b Unit for the Study and Treatment of Pain—ALGOS, Chair in Pediatric Pain URV-FG, Research Center for Behavior Assessment (CRAMC), Department of Psychology, Catalonia, Spain; c Unidad de Dolor, Hospital Obrero N.1-H. Materno Infantil, La Paz, Bolivia; d School of Medicine, Universidad Nacional Mayor de San Marcos, Lima, Perú; e Edwards Family Interdisciplinary Center for Complex Pain, Montréal Children's Hospital, Montreal, Canada; f Department of Anesthesia, McGill University, Montreal, Canada; g Department of Pain and Palliative Care, Federal University of Maranhao, Maranhao, Brazil

## Abstract

The Declaration of Lima on Pain in Childhood is a call into action to improve the care provided to children and adolescents with pain.

## 1. Pain in childhood

Pain is a common experience through the entire childhood from preterm babies to adolescents. For example, as far as acute pain is concerned, research has shown that newborns are subjected to numerous painful procedures in neonatal intensive care units.^1,2^ In addition, research has also shown that the prevalence of moderate-to-severe pain is high while they are in hospital, independently of the medical or surgical condition.^5^ Similarly, research on chronic pain has shown that prevalence is not only high^7^ but also increasing.^11,12^

Pain can have a severe effect on the life of children and adolescents and can become chronic. Patients with chronic pain can undergo significant physical, cognitive, and emotional problems.^6,8,9^ Moreover, chronic pain is a burden not only for the children who are afflicted but also for all those around them.^13^ Therefore, chronic pain becomes not only an individual health problem but also a social and economic one.^4^ In fact, the World Health Organization regards chronic pain itself as a disease,^14^ which has been conceptualized as a public health problem.^10^

## 2. Does pain in childhood matter?

Despite all the above, pain in children and adolescents is still mostly unrecognized, understudied, and undertreated.^3^ This seems to be the case in the Ibero-American region (a region including the countries in the Iberian Peninsula and in Central and South America), where, for example, lectures or presentations related to pain in children and adolescents at meetings of national pain societies or advocacy actions are almost nonexistent. On the basis of the observed needs in relation to the treatment, education, research, and advocacy on pain in childhood in the Ibero-American region, it was decided to promote the Declaration of Lima.

## 3. The Declaration of Lima on pain in childhood

The *Declaration of Lima on Pain in Childhood* is based on and indebted to the Declaration of Montreal, a document that emerged out of the International Summit on Pain organized by the International Association for the Study of Pain, in 2010. At that time, delegates proclaimed that most countries managed pain inadequately and declared that access to pain treatment is a human right. The *Declaration* was also inspired by the work done by the Ibero-American Research Network on Pediatric Chronic Pain (*Red Iberoamericana de Investigación en Dolor Crónico Infantil*).

The Declaration of Lima on Pain in Childhood is a call into action, a must needed step in the direction of helping to improve the care provided to children and adolescents with pain. It aspires to facilitate and promote a collaborative space in which patients, associations, researchers, clinicians, members of the industry, and legislators recognize needs and demands and help to improve the recognition and treatment of pain in the childhood in the Ibero-American region and the world. The motto underlying the Declaration of Lima is “Caring for children and adolescents with pain and their families is an inexcusable duty.”

## 4. The Declaration: a decalogue of proposals

In the framework of the 14th Latin American Congress on Pain (FEDELAT), the 18th Ibero-American Meeting on Pain and the 19th Peruvian Congress on Pain, we have drawn up the following decalogue under the title of the *Declaration of Lima on Pain in Chidhood*.

(1) Pain, and particularly chronic pain, must be conceptualized as a transversal pathology and not as a mere symptom associated with another pathology.
(2) Pain is a biopsychosocial experience. It is of such complexity that it should be managed by an interdisciplinary, not just a medical, approach.
(3) Children and adolescents in pain have the right to have their pain acknowledged and not be stigmatized.
(4) The right to pain management is nonnegotiable. Children and adolescents have the right to receive the best treatment available from expert professionals. Not facilitating access to pain management not only aggravates suffering unnecessarily but also discriminates and is unethical.
(5) To ensure that everyone has access to appropriate pain management, pediatric pain should matter, and all agents involved in facilitating treatment should be compelled to take all necessary actions to provide treatment and relief.
(6) Considering the legal scope of their power and authority, national governments must be responsible for (1) legislating and making plans to promote educational programs for health professionals that include, among other subjects, pain management; (2) enabling young people in pain to access the best possible treatment; (3) running programs to raise awareness and educate people about pediatric pain and its treatment; and (4) providing resources to adequately fund research on pediatric pain.
(7) Considering the legal scope of their power and authority, health institutions must be responsible for setting up systems to give young people in pain access to the best possible treatment.
(8) Considering the legal scope of their power and authority, health care professionals must be responsible for using empirical findings to provide the best treatment.
(9) Considering the legal scope of their power and authority, professional and scientific societies must be responsible for ensuring that their congresses and symposiums have pediatric pain-related content so that researchers and clinicians dedicated to the study and treatment of pediatric pain can engage in training and update their knowledge.
(10) Children and adolescents in pain and their families have a voice. This voice is of immense value and is a key tool for moving forward. It should be heard and incorporated into the drafting of laws, training programs, awareness campaigns, research promotion, and the planning of scientific conferences. Every step forward should be taken with the collaboration of those who experience the problem in the first person.

## 5. The Declaration: current situation and next steps

The presentation of the Declaration was on August 24, 2022. It was followed by a significant number of individuals who supported and signed on their behalf or, collectively, on behalf of the associations and societies which they represented. As promoters of the Declaration, we expect that it will serve to improve the situation of pediatric pain management in the region and promote the collaboration between stakeholders.

The Declaration has already served to plan webinars addressing pain in childhood in the region and pave the inclusion of pediatric pain-related contents in the Latin American Congress on Pain. Moreover, a working group has been ensembled to identify short-term and long-term needs and favor the collaboration between all the stakeholders: patients, health care professionals, researchers, professional societies, legislators and health managers, and industry.

Currently, the Declaration items are available in Arabic, Catalan, English, French, Portuguese, and Spanish at https://www.dolorinfantil.urv.cat/en/. Interested individuals and groups/associations/societies in showing their support for the Declaration can continue to do so with their signature at the following address: https://www.dolorinfantil.urv.cat/en/declaracio-de-lima/. A short video showing the presentation of the Declaration is available at https://www.youtube.com/watch?v=NH2-jb-Y8Do&ab_channel=SociedadEspa%C3%B1oladelDolorSED.

**Figure 1.**
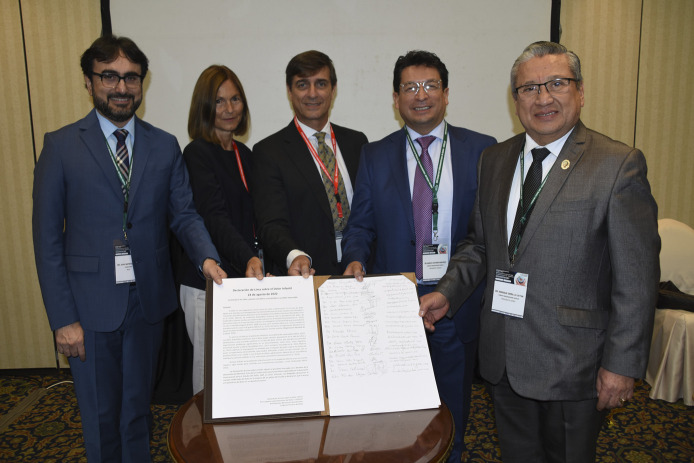
Showing the declaration and some of the signatures.

The picture accompanying this *Perspective* are of a key moment in Lima showing the Declaration and some of the signatures (Fig. 1).

## Disclosures

The authors have no conflict of interest to declare.
